# A Critical Review on Tropical Fruits Seeds as Prospective Sources of Nutritional and Bioactive Compounds for Functional Foods Development: A Case of Indonesian Exotic Fruits

**DOI:** 10.1155/2020/4051475

**Published:** 2020-03-18

**Authors:** Andri Cahyo Kumoro, Misbahudin Alhanif, Dyah Hesti Wardhani

**Affiliations:** ^1^Master of Chemical Engineering Study Program, Department of Chemical Engineering, Faculty of Engineering, Universitas Diponegoro, Semarang, Indonesia 50275; ^2^Institute of Food and Remedies Biomaterial, Department of Chemical Engineering Faculty of Engineering, Universitas Diponegoro, Semarang, Indonesia 50275

## Abstract

Some tropical countries in the Southeast Asia are rich in exotic fruits with worldwide acceptance, such as mango, orange, snake fruit, durian, jackfruit, rambutan, and avocado. In addition to their abundant production, those fruits are also currently gaining expansive distribution and marketing due to their promising advantages to human well-being. Surprisingly, their by-products, especially the seed kernel and peel, which account about 10–35% of their weight also offer high nutritional and functional potentials. This work exhibits the nutritional and bioactive compositions of the seeds of eight tropical exotic fruits, which are analyzed for their possible application as sources of functional food and environmental points of view. The seeds contain essential bioactive components, such as polyphenols, flavonoids, phenolic acid, and carotenoids, that exhibit excellent antioxidant activity, fats that have remarkable physicohemical properties (free of trans-fatty acids), and a high protein content. After a proper reduction of antinutritional contents, seed powders which contain carbohydrate, protein, and premium lipids or specific extracts with excellent functional properties can be obtained. However, further research should be carried out to determine the profiles of the nutritional and bioactive components in different seed types, their bioavailability, and their efficacy. Extensive researches with the industrial parts should also be performed to valorize the nutritional and functional potentials of these exotic fruit seeds.

## 1. Introduction

As the largest archipelago nation in Southeast Asia with 8.3 million km^2^ total area, Indonesia comprises 1.9 million km^2^ of land and is surrounded by 6.4 million km^2^ of sea [1]. Although the total area is only within 1.3% of the earth's, its tropical climate has made Indonesia one of the world's richest countries in terms of biodiversity. In fact, Indonesia is the home of at least 30,000–40,000 species of seed plants (spermatophyta) with 55% of them being endemic plants [2, 3]. The biodiversity also incorporates a variety of fruits, in which the Indonesian Agency for Research and Development, Ministry of Agriculture, the Republic of Indonesia, has reported that more than 592 species of fruit exhibit a large potential market [4].

A fruit is a type of plant, which humans and animals generally consume as their food source. Aside from energy, humans also require nutrition and various bioactive compounds from fruits, which demonstrate many health benefits. In addition, the fruits can be beneficial in lowering the risks of specific illness and functional declines related to ageing. Based on the data from the Statistics Indonesia (2017), there are 22 commercial fruits available in the Indonesian market which are considered as exotic fruits. Based on the annual production capacity, it can be concluded that there are ten major fruit commodities in Indonesia, namely, banana, mango, orange, pineapple, snake fruit, papaya, durian, jackfruit, rambutan, and avocado (Table 1). Although almost every province in Indonesia contributes to their production, Java and Sumatra are reported to be the leading producers of these fruits with more than 70% of all, i.e., banana (84.17%), mango (80.70%), and orange (73.21%).

Some exotic fruits, specifically their pulps, are commonly eaten when ripe (i.e., banana, avocado, and rambutan) or when immature (i.e., mango). Most of them are eaten fresh. However, some particular types of fruits are consumed as processed foods, which are made from the pulp to overcome excess production during harvest season [5]. In addition, some potential utilizations of fruit by-products had been reported in the literature through the isolation of their specific bioactive compounds for further application in nutraceutical and food supplements, functional food, and pharmaceutical products [6]. O'Shea et al. [7] reported that about 35–60% of the fruits are disposed of as by-products after being processed, especially for the case of their seeds. The summaries of the annual production of Indonesian exotic fruits, their seed percentage in the fruit, and kernel percentage in the seed are tabulated in Table 1.

Table 1 indicates that except for banana, orange, and pineapple, most Indonesian exotic fruits contain seeds of about 5–45% of their total weight. Except for banana, pineapple, and papaya, the seeds also contain kernels that are 40–98% of their weight. Since most of the selected Indonesian exotic fruit kernels are edible after processing, Table 1 also demonstrates their broad potential for various applications. In order to explore wider potential applications of the selected Indonesian exotic fruit kernels in the production of functional food and nutraceutical products, qualitative and quantitative analyses of their nutritional and bioactive components are very crucial. The primary objective of this manuscript is to elaborate and summarize the potential and advantages of the Indonesian exotic fruit seeds in terms of their nutritional composition and functional properties. Accordingly, this manuscript also elaborates on the nutraceutical components and medicinal properties of the Indonesian exotic fruit seeds. This study was conducted on eight various types of fruit seeds, which are selected based on their high annual whole fruit and kernel seed productions.

## 2. Indonesian Exotic Fruit Seeds

### 2.1. Taxonomy

The scientific classifications of eight selected Indonesian exotic fruits are obtained from the USDA PLANTS Database as shown in Table 2 [17].

### 2.2. Mango Seed

**Figure 1:**
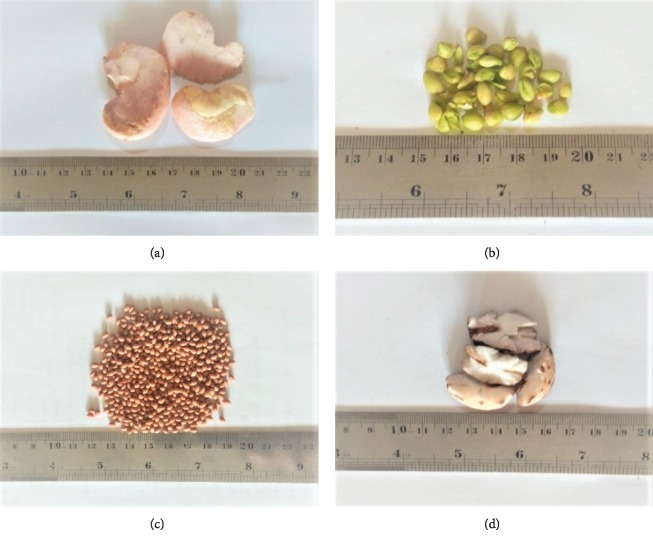
Fruit seed kernels: (a) mango, (b) sweet orange, (c) papaya, and (d) snake fruit.

Naturally, mango (*Mangifera indica*) contains a single seed in two pieces (dicotyledone), which comprises a thick-woody outer shell (endocarp) surrounding the kernel (the right seed) [18]. However, mango seeds (Figure 1(a)) can be either monoembryonic (result seedlings) or polyembryonic (result various plants). In fact, most cultivated varieties of mango in Indonesia, Malaysia, Myanmar, Thailand, and the Philippines are polyembryonic [19]. Depending upon the cultivar, the mango seed may account for 10 to 25% of the total fruit weight, while the kernel contributes 45 to 85% of the seed weight, or about 20% of the whole fruit weight [9].

### 2.3. Sweet Orange Seed

Jeruk Pontianak (*Citrus nobilis Loureiro* var. *microcarpa Hassk*) is the most popular citrus fruit plant species available in Indonesian markets. Jeruk Pontianak or Pontianak orange is affiliated with the mandarin orange group. Morton [20] suggested that *Citrus nobilis* is more likely to be the crossbreed between sweet orange (*Citrus sinensis*) and mandarin orange (*Citrus reticulata*). Pontianak orange is the dominant cultured citrus cultivar in Indonesia owing to its large production yield, simplicity of cultivation, and being the most preferred by the local residents [21]. It has slim, relatively shiny, and yellowish green-colored peel. The fruit is sized from 5.5 to 5.9 cm in diameter. The flesh is orange in color and carries plenty of extremely sweet juices. The history of Pontianak oranges in Indonesia is thought to have begun in 1936, when they were first planted in surrounding towns alongside Pontianak of West Kalimantan province in Indonesia by local agrarians [21]. Unfortunately, there was a serious devastation of this cultivar as a consequence of Indonesian government policy and disease infection in the past few decades.

In general, orange seeds (Figure 1(b)) exhibit greenish to pale whitish color, with a flattened and angular shape. They are polyembryonics, which are mostly zygotics or nuclear. The zygotic embryos are the result of pollination of the ovary through sexual reproduction and hence are not always equal in horticultural qualities to their parent trees. On the other hand, the nuclear embryos are purposely derived whole from the mother plant and exhibit close characteristics similar to their parent trees.

### 2.4. Papaya Seed

Papaya (*Carica papaya*) is a member of the most popular fruit plants from the genus Carica and is widely cultivated all over the tropical areas. The flesh of papaya is mainly consumed fresh, whereas its industrial application is mostly for the preparation of candies, jam, jelly, and pickles [22]. The papaya fruit seeds (Figure 1(c)), contributing close to 20% of its total weight [23], can be prospectively worthwhile in view of their nutritional and functional components. Moreover, the seeds are eatable and sometimes can be used as a replacement of black pepper due to their spicy and pungent flavor [24]. Papaya flesh has been used as a traditional therapeutic remedy due to its fabulous curing abilities, while the seeds are also recognized to exhibit a number of nutritional and health benefits. The fruit contains various phytochemicals, especially carotenoids and polyphenols [25]. The emerging potential of the antioxidant in the papaya peel and seeds may contribute to the manufacture of functional foods and nutraceuticals in the foreseeable future utilizing these papaya wastes [26]. However, there is scarce information on this relatively underutilized seed despite its importance. Hence, papaya seeds, which constitute close to 30–35% of the waste derived from the fruit, are usually discarded.

Even though papaya seeds contain 27.3%–28.3% protein, 28.2%–30.7% lipids, and 19.1%–22.6% crude fibers, they have yet to be commercially exploited. Almost all of the seeds are produced in the form of lees and disposed of as a horticultural by-product during the fruit processing, triggering environmental issues.

Papaya seeds are rich in oil content (13.9–30.7%), which mainly consists of monounsaturated fatty acids and nutraceuticals, such as the phenolics tocopherol and carotenoid. Furthermore, papaya seed oil (PSO) was reported to be persistent towards oxidation, which can be transformed into an unconventional kind of cooking oil consisting of exceptional health benefits in food applications [27]. This offers the perception of minimizing environmental pollution and causing waste seeds to become rewarding.

### 2.5. Snake Fruit Seed

Snake fruit (*Salacca edulis* Reinw) is among the most favorite fruit plant species in the palm or Arecaceae family. In Indonesia, Malaysia, Brunei, and Singapore, it is called “salak,” which is similarly spelled as “sa laka” or “she pi guo zong” in China. But the Thais and Burmese call it “rakam” and “yingan,” respectively [28]. Indonesia has several varieties of snake fruit, which includes Pondoh, Nglumat, Bali, Swaru, Enrekang, and Gula Pasir [29]. Physically, the snake fruit is like a triangular-shaped stone which is rather round or inverted, pointed at the base and rounded at the end, and 2.5–10 cm long. This fruit structure is wrapped in yellow brown to brown shiny red scales orderly organized like tiles, with a lot of sharp tiny thorns that break easily at the ends of each scale. Some creamy yellow to whitish edible thick pulps with sweet, sour, or stomach taste are located in the middle of the fruit (sarcotesta) [30]. Snake fruit may contain 0–3 brown to black seeds of 2–3 cm long, which are covered by hard and thick outer shells (endocarp) enclosing the kernel.

According to Supriyadi et al. [11], snake fruit seeds (Figure 1(d)) contain 15–20% of the total weight of fruits with the kernels contributing 60–75% of the seed weight. Surprisingly, the seed kernels of young “Pondoh” snake fruits (one of varieties from Indonesia) can be eaten directly (Figure 1(d)) [31]. In addition, after a carefully controlled pyrolysis, snake fruit seeds are recently used in coffee drink preparation in Bali due to their specific taste, aroma, and health benefits.

### 2.6. Durian Seed

**Figure 2:**
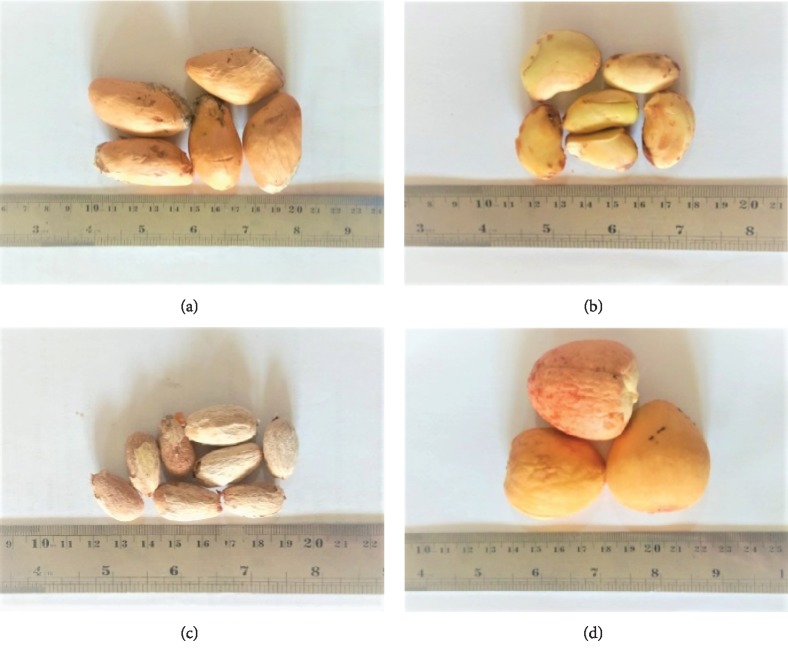
Fruit seed kernels: (a) durian, (b) jackfruit, (c) rambutan, and (d) avocado.

Durian (*Durio zibethinus*) is a popular seasonal fruit from Southeast Asia (Indonesia, Malaysia, Thailand, the Philippines, and Brunei Darussalam). However, today's agricultural technology has enabled farmers to produce durian fruit almost all the year round. Durian is also well known as the “king of fruit” by people in this area due to its unique and preferable taste and aroma. Depending on the cultivar, durian fruit pulp, which is the edible portion, is sweet or bitter-sweet in taste and is either white, yellow, golden yellow, pink, or red in color. The fruit consists of sharp thorny skin, pulp, and seeds. The pulp can be directly eaten as fresh fruit or further processed into various types of food products, such as preserved durian, dried durian chips, and frozen durian. Some sharp egg round seeds are located inside the edible pulp and are enclosed by a thin, yellowish colored skin (Figure 2(a)) [32]. Unfortunately, literature studies have concluded that durian fruit culture produces approximately 67–70% of wastes, in the form of seeds (20–25%) containing 95–98% of kernel seeds and about 75% of the nonedible rind or shell [33].

### 2.7. Jackfruit Seed

Jackfruit (*Artocarpus heterophyllus* Lam.) is a tropical fruit native to India, Thailand, and Indonesia. However, it can be found easily in many parts of Asia and Northern Australia and even in Africa and South America. Similar to durian, today's agricultural technology has helped farmers to harvest jackfruit almost all year round. The weight of a ripe jackfruit can reach 2 to 36 kg with 22–90 cm in length and 13–50 cm in diameter. The ripe fruit is composed of neatly arranged fleshy sweet bulbs with banana flavor, which may be crispy or soft in texture and yellow to brownish in color. The shape of ripe fruit seeds varies from round to oval to long. Approximately 100 and 500 seeds are covered by the bulbs, which represent 18–25% of the total weight of the fruits with the kernels amounting to 90–95% of the seeds, while the pulp accounts for 30% of the fruit weight. The seed kernels are white and usually sized between 2 and 3 cm in length and are 1–1.5 cm in diameter. The seed kernel is coated by an outer thin white shell layer and light brown hull (Figure 2(b)) [34]. In Indonesia, jackfruit seeds are commonly consumed as boiled or roasted by people in the rural areas, while some other people use them as vegetables.

### 2.8. Rambutan Seed

Rambutan (*Nephelium lappaceum*) fruit is well renowned for its appealing soft hairy skin varying in color from either green, red, yellow, or orange yellow [35]. The fruit is normally sized at around 3–6 cm in length and 3–4 cm in diameter. The fruit is composed of peels which are 45.7%, pulp which is 44.8%, seed which is 9.5%, and embryo which is 6.1% of the total weight [15]. The edible portion of the fruit pulp is rich in sugars, organic acids, and ascorbic acid [36]. The seed is light brown and sized within 2–3 cm in length (Figure 2(c)) [37]. After the fruit pulp is processed into jelly, jam, and juice, the rind and seed are discarded. Darajati et al. [2] reported that rambutan seeds are slightly bitter in taste and have narcotic properties given the fact that they contain alkaloids. In addition, the testa is found to contain tannins and saponins. Although fresh rambutan seeds are believed to be toxic, some folks in the Philippines have been reported to safely consume the roasted ones [15].

### 2.9. Avocado Seed

The avocado (*Persea americana* Mill) fruit is pear-shaped, often almost necked, oval, or closely rounded. They may be 7.5–33 cm long and approximately 15 cm wide. The skins or peels may be yellow green, deep green, reddish purple, or such a dark purple as to look almost black and are occasionally freckled with many small yellow dots [38]. The fruit is composed of pericarp (hull), mesocarp (pulp), and endocarp (seed). Although in Latin America the fruit pulps are merely utilized in essential oil production [39], Indonesian people use the fruit pulps for the preparation of local beverages, such as ice cream, avocado juice, or a complementary component of “*Es Teler*,” thus leaving the seeds, peels, and exhausted pulp as residues. Therefore, a huge amount of solid seed residues that correspond to approximately 21–30% of the fruits is commonly discarded [16]. The kernels account between 95 and 98% of the seeds and contain around 20% of starch [40].

The seed are commonly round, conical, or ovoid, 5–6.4 cm long, hard and heavy, and creamy white in color but covered in two brown, thin, papery seed coats often bonding to the pulp cavity, from which the right seed comes out easily. As shown in Figure 2(d), the seed is composed of a very thin shell (endocarp) that wraps the kernel. Unfortunately, some avocado fruits are seedless as a result of a lack of pollination or other cultivating parameters [20].

## 3. Nutritional Potentials of Indonesian Exotic Fruit Seeds

In addition to the peel, core, residual pulp, stem, and leaves, fruit seeds can be considered as the prominent by-product of fruit processing industries. Indeed, the seeds found in any fruits vary in their sizes and weights [9]. As mentioned in Section 1, Indonesia has eight candidates of exotic fruits with high annual productivity, such as mango, orange, snake fruit, papaya, durian, jackfruit, rambutan, and avocado. These fruits offer great potential as main sources of functional foods. Several investigations had been carried out on the nutritional content of eight Indonesian exotic fruit seeds, which found that they exhibit different nutritional contents when the fruits are cultivated with different varieties and growing conditions. The nutritional composition of the Indonesian exotic fruit seeds (on a weight basis), such as the moisture, carbohydrate, protein, lipid, fiber, and ash contents, is tabulated in Table 3.

The data presented in Table 3 shows that the carbohydrate content of mango seed kernels is the highest in comparison to other nutrients [41] and is being comparable to that in durian seeds. The carbohydrate level in mango seed kernels suggests that it can be used as energy source foods. In some parts of India, the kernel starch is consumed as a staple food during famine [42]. The protein content in mango seeds is on par with papaya seed kernels. Even though the mango seed kernel is found to possess a low protein content, it exhibits an excellent quality [43]. The ash content of mango seed kernels is comparable with that of rambutan's and indicates a high level of sodium, calcium, magnesium, phosphorus, and potassium [44]. Fahimdanesh and Bahrami [45] suggested that the mango seed kernel is a potential source of lipid. Depending on the cultivars, the neutral fat content of the mango seed kernel varies from 95.2 to 96.2%, phospholipids from 2.7 to 3.3%, and glycolipids from 1.1 to 1.4% [46]. Triglycerides contribute to the main portion of the neutral fats and accounted for around 93.7 to 96.4%. The fats of mango seed kernels contain roughly 44–48% of saturated fatty acids (mainly stearic acid) and 52–56% unsaturated (primarily oleic acid). Due to its rich content of palmitic, stearic, and oleic acids, the mango kernel lipid demonstrates some advantageous physicochemical properties [47], including its prospective utilization as a replacement for cocoa butter in the bakery industries [48]. Being rich in nutrients and nontoxic (free from toxic substances, such as cyanogen), the fat of mango seed kernels could be directly used to substitute for any food lipid without deleterious influences on the quality [46].

Table 3 provides information that the lipid represents the major nutrient in the sweet orange seed followed by carbohydrate, crude fiber, and protein. Because of its high lipid and carbohydrate contents, orange seed flour exhibits high energy values in the range of 609 to 614 kcals/100 g [49]. Therefore, they are not prone to microbial degradation or germination. Sweet orange seeds can thus be an essential source of cooking oil. Thus, the potential of orange seed usages in human and animal foods looks promising. A relatively high crude fiber content suggests that sweet orange seeds can be utilized as a promising source of dietary fiber. Sweet orange seed kernels possess a high content of ash, in which potassium, calcium, sodium, and iron are the major minerals. The high potassium/sodium ratio (22 : 1), observed in the dehulled seeds, is preferred because most of today's human diets are low in potassium but high in sodium [56]. The low level of sodium in sweet orange seeds makes them suitable for use in sodium-restricted diets. The availability of iron from food has always been of interest to nutritionists. The iron in orange seeds, even though low, may be well utilized. If calcium in the seeds is in the available form, it would serve as a good source of organic calcium to consumers [49]. The moisture content of the seed kernel is low and is comparable to that of papaya and rambutan seeds.

Literature survey revealed that research on snake fruit seeds is still very scarce causing limited information of their potential applications. Kusumo et al. [50] reported that the snake fruit seed kernel is rich in carbohydrate, fiber, and moisture. However, it is strongly attached to a woody hard shell and the processing of snake fruit seed becomes very difficult. The carbohydrate of snake fruit seeds consists of 28.98% cellulose and 59.37% glucomannan [57]. One of the most creative efforts is by using the roasted whole snake fruit seeds as a substitute for coffee. The snake fruit seed coffee contains 6.24% moisture, 80.98% carbohydrate, 2.95% lipid, 6.34% protein, 3.49% ash, and 0.207% caffeine [58]. In addition, glucomannan of the snake fruit seed kernel can be used in the edible film, adhesives, insulation, foods, paint, cosmetics, and pharmaceutical industries [59].

The data tabulated in Table 3 shows that papaya seed kernels contain a comparable amount of carbohydrate with mango and durian seed kernels. Unfortunately, papaya seeds are spicy and peppery causing these food materials to be less palatable [43]. Fortunately, the papaya seed kernel has superior protein, lipid, and fiber contents than any other fruits. Due to their rich lipid content, papaya seeds could be economically engaging for industrial utilization similar to other traditional oilseed producer plants such as corn and soybean. Puangsri et al. [60] reported that the main fatty acids of the papaya seed oil were oleic (76.9%), palmitic (13.4%), stearic (4.6%), and linoleic (3.2%) acids. Besides, trace amounts of palmitoleic, myristic, margaric, arachidic, eicosenoic, lauric, and linolenic acids were also observed. The lower linoleic acid content in papaya seed oil exhibited stronger stability against oxidation than any other seed oils [61]. Further, Malacrida et al. [62] also proved that papaya seed oil showed excellent strength against oxidation without any addition of artificial antioxidants. The regular intake of high amounts of this particular type of oils could induce a reduction in the risk of coronary heart disease. Moreover, high-oleic oils possess satisfactory stability to be utilized in extremely demanding food processing such as frying. Other edible applications are in the field of spatter oils for snacks, crackers, cereals, and bakery products, where the oil is utilized to preserve product quality and enhanced palatableness. The high content of unsaturated fatty acids would enable the oil as a plausible replacement for other highly unsaturated oils. Nevertheless, prior to being utilized for food product manufacture, toxicological investigations are required to be performed to ensure whether or not this oil is harmful for human consumption [60].

Table 3 reports that the carbohydrate content of durian seed kernels is comparable with mango seeds. Amin and Arshad [13] reported that durian seed flour can be introduced into food products such as cake, cookies, soup, and tempura, because it is healthful and rich in dietary fiber. Besides, the existence of hydrocolloids and starch in durian seed flour enables it for use as a thickening agent. Durian seed kernels exhibit low protein content, which is comparable with that of orange's. This suggests that durian seed kernel flour (DSKF) is not appropriate for the preparation of bread; however, it could be proper for the preparation of cookies or cakes. Fortunately, the lack of gluten in DSKF will be suitable for the preparation of food for people with celiac disease. The lipid content of durian seed kernels is low and comparable to that of avocado's. Despite its higher fiber content, the durian seed kernel is less gritty in comparison to whole meal wheat flour. Therefore, in terms of lipid and fiber contents, the durian seed kernel shows its superiority as a healthier food material compared to wheat flour. The ash content of the durian seed kernel is within the same range of most of other fruit seed kernels (0.90–2.50%).

The carbohydrate content of jackfruit seed kernels shown in Table 3 is high and on par with that of sweet orange, rambutan, and snake fruit seed. This suggests that if added to human and animal foods, these seeds could be used as a reasonable source of energy. Based on its shear and temperature effects, the chemically modified jackfruit seed starch possesses wide potential applications in various food and pharmaceutical applications [63, 64], such as confectionery filling, thickener and stabilizer in chili sauce and gum candies, and binder in pharmaceutical tablets as well as a mucoadhesive agent in drug delivery devices [65]. Surprisingly, jackfruit seeds are also rich in protein and the protein contents are higher than those of animal origin such as cattle and seafood [66], while the lipid content is the lowest compared to other fruit seeds. Therefore, the processed seeds like chip and flour will be less vulnerable to fat-related forms of degradation [67]. The lipid is steadily liquid at room temperature and could be advantageous for nutritional and industrial applications. The major amino acids in jackfruit seeds are methionine, leucine, alanine, and threonine, while the fatty acids are linolenic and linoleic acids. Fructose and sucrose are the main sugars in jackfruit seeds [68]. In addition, jackfruit seed gum can be used as a functional food ingredient, specifically as a soluble dietary fiber supplement to food products [69]. Azeez [68] concluded that the roasted jackfruit seeds can be used as an alternative raw material for the manufacture of healthy snack products due to their high dietary fiber content. The optimum roasting conditions of jackfruit seeds are reported to be at 153.4°C and pH 6.34 for 34.4 minutes. The flowery, green, pungent-sulfurous, sweet caramel, and woody aromas are more pronounced in the roasted seeds compared to the raw seeds due to the presence of monoterpenes, esters, ketones, alkanes, aldehyde, and alcohols [68]. The seed kernels exhibit comparable mineral content with other fruit seed kernels and are found to be favorable sources of minerals. Potassium was the dominant mineral element which is 2470 ppm followed by calcium, magnesium, and sodium. In addition, the seed kernel also contains a reasonable quantity of iron, which is 148.50 ppm [70].

Table 3 clearly presents that the rambutan seed is rich in carbohydrate, protein, and lipid. Although the rambutan seed contains a moderate content of protein, the defatted seed flour exhibits a high-quality protein due to the existence of the most essential and nonessential amino acid profiles [71]. The relative amounts of essential amino acids present in the rambutan seed in decreasing order are lysine, leucine, valine, isoleucine, phenylalanine, methionine, and histidine [71]. However, nonessential amino acids are present in the descending order of relative percentages as glutamic acid>glycine>aspartic acid>arginine>serine>threonine>alanine>tyrosine>proline>cysteine [71]. The major fatty acids in the lipid of rambutan seed kernels are palmitic acid (4.36–4.86%), stearic acid (5.93–7.49%), oleic acid (37.91–40.15%), and arachidic acid (36.14–36.77%) [15, 54]. The composition of the lipids of rambutan seed kernels is closely similar to that of cocoa. Even though having slightly different physical characteristics, these lipids were reported to be a suitable substitute to cocoa butter for confectionery products [72]. Based on the brine shrimp lethality test, Chai et al. [73] confirmed that the roasted rambutan seed powder is not toxic and is safe for human consumption. A blend of 30% mass of rambutan lipid and 70% mass of cocoa butter can result in characteristics similar to cocoa butter but with a higher viscosity [74]. This observation suggests that the rambutan seed kernel can be converted into a cocoa-like powder product after being subjected to 8 days of fermentation [36, 75], drying at 60°C for 48 hours in a convection oven, and roasting at 131°C for 25 min [76]. Trimethylpyrazine and tetramethylpyrazine were identified in the roasted rambutan seed, which is similar to that of commercial cocoa powder. The moisture content of the rambutan seed kernel is low and leads to safe storage even without further drying. However, Chimplee and Klinkesorn [77] suggested that 65°C is the optimum drying temperature to reduce the seed moisture and improve the fatty acid extraction yield. Attractively, a higher content of arachidic acid makes the lipid suitable for food application without worrying about an extensive hydrogenation process, specifically when dealing with autoxidation. Considering its advantageous nutritional components, the rambutan seed can be utilized as a promising source for future applications in the food industry.

Table 3 shows that avocado seeds possess the highest carbohydrate content compared with any other fruit seeds but with low lipid content. Therefore, avocado seed kernel flour can be considered as a healthy raw material and can be used as a good substitute to wheat flour and other commercial flours and starches, such as potatoes, sweet potatoes, and cassava in food applications [38]. The ash content of avocado seed, which indicates the total amount of minerals present within food, is comparable with jackfruit, snake fruit, and rambutan seeds. However, because the protein and fiber contents of avocado seed kernels are low, an enrichment of protein and fiber from other sources is necessary. In addition, as the moisture content of the avocado seed is high and comparable with mango, snake fruit, durian, and jackfruit seed, a rapid drying system will be required to prevent the seed from germinating or decomposing.

## 4. Bioactive Components of Selected Tropical Fruit Seeds

Some reports on the identification of bioactive compounds on some tropical exotic fruit seeds are listed in Table 4. Due to the scarcity of the data related to nutraceutical compounds in Indonesian exotic fruit seeds, in this study, some of them are taken from the results of research in the regions around Indonesia, especially Southeast Asia.

The total polyphenol content of Indonesian exotic fruit seeds varies from 212 to 839 mg gallic acid equivalent (GAE) per 100-gram seeds [78–81] (Table 4). Almost all of the exotic fruit seeds in this study contain polyphenols, except snake fruit seed. Polyphenols contribute numerous medicinal properties such as antioxidant, anti-inflammation, antiviral, antithrombogenic, and anticarcinogenic actions [82]. In addition, exotic fruit seeds also contain flavonoids, which are a group of phenolic compounds and are able to enhance immunity, antiallergy, anticancer, antioxidant, anti-inflammation, antiplatelet, inhibition of tumor growth, antivirus, antimicrobial, antispasmodic, diuretic, and vasoprotection [80]. The sweet orange seed has the highest flavonoid content compared to other fruit seeds.

In this study, the phenolic acids in the exotic fruit seeds are grouped into hydroxycinnamic acid (caffeic acid, coumaric acid, ferulic acid, chlorogenic acid, and homogentisic acid) and hydroxybenzoic acid (gallic acid, kaempferol, catechin, cyanidin-3-glucoside, and tangeretin) derivatives. They show as anticancer and anticarcinogenic activities [81]. The main phenolic acids found in Indonesian fruit seeds are caffeic acid, coumaric acid, ferulic acid (i.e., mango, sweet orange, and papaya seeds), gallic acid (i.e., avocado seed), and kaempferol (i.e., mango seed). The snake fruit, durian, jack fruit, rambutan, and avocado seeds also perform some antiproliferative activities against cancer cell lines [82]. In addition, sweet orange, papaya, and rambutan seeds exhibit high carotenoid content such as *β*-carotene in sweet orange seeds.

This article also reviews the capabilities of tropical exotic fruit seeds to hinder the growth of cancer cell lines in the human lung (A549), breast (MCF-7), liver (HepG2), and colon (HT-29) [83–85]. As seen from Table 4, snake fruit, durian, jackfruit, rambutan, and avocado seeds showed relatively powerful inhibitory capacities towards all the four cancer cell lines. In particular, snake fruit and rambutan seeds displayed the strongest inhibitory activity against the growth of A549, HepG2, and HT-29. Snake fruit, rambutan, and avocado seeds demonstrated the strongest inhibitory activity against the growth of MCF-7. For sweet orange and papaya seeds, the antiproliferative capacity to inhibit the growth of cancer cells is not known. This can be an opportunity for further study, considering that sweet orange and papaya seeds also have flavonoids which are one of the anticancer agents.

## 5. Technological Perspective and Future Trends

The tropical exotic fruit seeds exhibit their extensive potentials as a natural source of ingredients and additives to create added value in modern foods. The potentials include the nutritional and bioactive components for further uses in the food, cosmetics, and pharmaceutical manufactures. At the moment, several technologies for utilizing fruit seeds in the form of flour and specifically extracted compounds have been developed. Being highly rich in lipid, protein, and carbohydrate content with inexpensive costs should promote tropical exotic fruit seeds' potential uses in food industries [105]. Some specific fruit seed extracts also can be utilized in the food industry for numerous applications, such as as a preservative, flavor enhancer, and colorant in the confectionery and bakery items.

Cuq et al. [106] proposed a feasible processing method to convert mango seed kernels into flour which thereby offers extensive technological applications. Powder food materials are more convenient to convey, store, process, dose, and utilize. Fortification of mango seed kernel flour in food is a smart way to provide adequate consumption of antioxidants, protein, and lipid required by consumers at a reasonable price. In addition, rambutan seeds with their high fat and oil content of superior quality can be used for cooking and soap manufacturing [37]. Avocado seeds can be used as antioxidant tea drinks and a culture medium. Snake fruit seeds would be highly valuable in the health or pharmaceutical industries due to their excellent activity in inhibiting cancer cell growth [83]. Durian and jackfruit seeds with their high carbohydrate and protein contents can be processed into flour or starch and further used in the manufacture of various food products. With high carotenoid, lipid, carbohydrate, and protein contents, papaya and sweet orange seeds can be good candidates for food fortification agents. Processing, extracting, refining, and utilizing specific compounds in fruit seeds with high nutritional and bioactive components can be a smart idea to utilize and add value to the fruit seeds.

## 6. Summary

This article colligates the nutritional and bioactive components of selected tropical exotic fruits seeds that have the highest annual productivity, including mangoes, sweet orange, snake fruit, papaya, durian, jackfruit, rambutan, and avocado, and proposes their potential utilization as raw materials or substitutes in food and pharmaceutical productions. The large annual productivity of these fruits has generated a vast amount of residue, especially the seeds. Scientific studies have revealed the existence of several polyphenols, flavonoids, phenolic acids, and carotenoid-derived compounds in Indonesian exotic fruit seeds that are useful to human health, and the role of these compounds as anticancer, antivirus, antiobesity, antidiabetes, antimicrobial, antioxidant, and anti-inflammation agents has been proven via in vitro and in vivo assays. Because some of these fruit seeds also carry large amounts of lipid, such as orange, rambutan, and papaya seeds, they also can be a good source of functional fats. The usage of fruit seeds in the beverage, pharmaceutical, nutraceutical, and functional food industries can not only solve waste problems but can also generate additional revenue for the fruit processing enterprises. Unfortunately, there are still many nutraceutical components of fruit seeds, especially snake fruit, papaya, and sweet orange, which are not known. In addition, information on the possibility of anticancer activity in papaya and sweet orange seeds also has not been found. Therefore, it is very important to perform further analysis of the nutraceutical components and their utilizations in order to provide comprehensive information to determine the suitable utilization of these seeds and their incorporation as ingredients in the food, food supplement, pharmaceutical, and cosmetics manufacturing. However, as wider areas of fruit seed potentials require further investigation, this review can be a beneficial resource for upcoming research activities.

## Figures and Tables

**Table 1:** The annual production of Indonesian exotic fruits in 2017 [8].

No.	Type of fruit	Production (ton)		Growth (%)	Seed content (%)	Kernel seed (%)	Kernel seed potentials (ton)	Functional food utilization potentials	Ref.
		2016	2017						
1	Banana	7,007,125	7,162,685	2.22	Seedless	0	0	No	
2	Mango	1,814,550	2,203,793	21.45	10–25	45–85	228,543.25	Yes	[9]
3	Sweet orange	2,014,214	2,165,192	7.50	1.80	71.43^a^	27,838.74	Yes	[10]
4	Pineapple	1,396,153	1,795,986	28.64	Seedless	0	0	No	
5	Snake fruit	702,500	953,853	35.81	30–45	90.16^a^	298,673.75	Yes	[11]
6	Papaya	904,284	875,112	-3.23	6.5–15	12.77^a^	12,213.56	Yes	[12]
7	Durian	735,423	795,211	8.13	20–25	95–98^a^	164,323.17	Yes	[13]
8	Jackfruit	654,914	656,583	0.25	8–15	90–95^a^	68,542.11	Yes	[14]
9	Rambutan	573,193	523,704	-8.47	4–9.5	39.10	14,474.93	Yes	[15]
10	Avocado	304,938	363,157	19.09	21–30	95–98^a^	82,200.74	Yes	[16]

^a^Found in this study through manual measurement.

**Table 2:** Scientific classification of the Indonesian exotic fruits.

	Mango	Sweet orange	Papaya	Snake fruit	Durian	Jackfruit	Rambutan	Avocado
Kingdom	Plantae	Plantae	Plantae	Plantae	Plantae	Plantae	Plantae	Plantae
Subkingdom	Tracheobionta	Tracheobionta	Tracheobionta	Tracheobionta	Tracheobionta	Tracheobionta	Tracheobionta	Tracheobionta
Super division	Spermatophyta	Spermatophyta	Spermatophyta	Spermatophyta	Spermatophyta	Spermatophyta	Spermatophyta	Spermatophyta
Division	Magnoliophyta	Magnoliophyta	Magnoliophyta	Magnoliophyta	Magnoliophyta	Magnoliophyta	Magnoliophyta	Magnoliophyta
Class	Magnoliopsida	Magnoliopsida	Magnoliopsida	Liliopsida	Magnoliopsida	Magnoliopsida	Magnoliopsida	Magnoliopsida
Subclass	Rosidae	Rosidae	Dilleniidae	Arecidae	Dilleniidae	Hamamelididae	Rosidae	Magnoliidae
Order	Sapindales	Sapindales	Violales	Arecales	Malvales	Urticales	Sapindales	Laurales
Family	Anacardiaceae	Rutaceae	Caricaceae	Arecaceae	Bombacaceae	Moraceae	Sapindaceae	Lauraceae
Genus	Mangifera	Citrus	*Carica*	Salacca	Durio	Artocarpus	Nephelium	Persea
Species	*Mangifera indica*	*Citrus nobilis*	*Carica papaya*	*Salacca zalacca*	*Durio zibethinus*	*Artocarpus heterophyllus*	*Nephelium lappaceum*	Persea americana

**Table 3:** The nutritional content of the kernel seed of Indonesian exotic fruits (% *w*/*w*).

No.	Type of fruit	Carbohydrate	Protein	Lipid	Fiber	Ash	Moisture	Ref.
1	Mango	18.94	4.40	5.37	0.84	1.81	51.5	[41]
2	Sweet orange	28.5	3.06	54.2	5.5	2.5	6.28	[49]
3	Snake fruit	23.09	4.22	0.48	15.81	1.56	54.84	[50]
4	Papaya	14.4	28.2	27.0	21.8	2.4	6.2	[51]
5	Durian	18.92	3.4	1.32	19.88	1.58	54.9	[52]
6	Jackfruit	25.80–38.40	6.6–7.04	0.40–0.43	1.00–1.50	0.90–1.30	51–65	[53]
7	Rambutan	41.18 ± 0.43	7.6 ± 0.14	38.0 ± 4.36	2.4 ± 0.32	1.22–2.26	9.6 ± 3.52	[54]
8	Avocado	41.1	2.5	1.0	3.7	1.3	50.4	[55]

**Table 4:** Nutraceutical potentials of Indonesian exotic fruit seeds.

Bioactive compound	Mango	Sweet orange	Papaya	Snake fruit	Durian	Jackfruit	Rambutan	Avocado	Efficacy and health benefit	Ref.	
*Phenolic compounds*										
Polyphenols (mg GAE/100 g dry seeds)	754 ± 24.0	212 ± 8.4	1945.48 ± 45.55	nd	367 ± 26.0	243 ± 27.0	305.0	839 ± 27.0	Antioxidant, anti-inflammatory, antiviral, antithrombogenic, and anticarcinogenic actions	[78–81, 86]	
Flavonoids (mg CE/100 g dry seeds)	870-950	8000	117.48 ± 15.54	nd	nd	2.03 ± 0.06	1330	47.97 ± 2.6	Boost immunity, antiallergy, anticancer, antioxidant, anti-inflammatory, antiplatelet, antitumor, antivirus, antimicrobial, antispasmodic, diuretic, and vasoprotection	[79–81, 86–89]	
Tannins (mg/100 g seed)	210-360	0	nd	nd	nd	0.06-0.229	0.15	nd	Accelerate the healing of wounds and anti-inflammation (swollen boils, hemorrhoids, frostbite, and burns)	[86, 87, 89, 90]	

*Phenolic acids*										
Quercetin (mg/100 g seed)	nd	nd	11 ± 1.00	0.13	nd	nd	nd	nd	Hypoglycemic activity by increasing insulin production in pancreatic *β* cells	[91, 92]	
Caffeic acid (mg/100 g seed)	89.43-326.9	1.1 ± 0.2	19 ± 0.00	nd	nd	UDL	nd	13.69 ± 1.27	Antioxidant and antibacterial activity in vitro and prevention of atherosclerosis and other cardiovascular diseases	[91, 93–95]	
Coumaric acid (mg/100 g seed)	39.65 ± 1.98	1.8 ± 0.2	13 ± 2.00	nd	nd	nd	nd	nd	Antioxidant, cardioprotective, antimelanogenic, antimutagenic, antiplatelet, anti-inflammatory, and immunomodulatory activities	[83, 91, 94]	
Ferulic acid (mg/100 g seed)	168.63-407.02	4.6 ± 0.7	26 ± 0.00	nd	nd	0.216	nd	0.087 ± 0.1	Hypoglycemic activity by increasing insulin production in pancreatic *β* cells	[91, 93–96]	
Gallic acid (mg/100 g seed)	3.84 ± 0.2	nd	nd	UDL	nd	1.105 ± 0.1	2.84 ± 0.33	6200 ± 230	Antioxidant; anti-cardiovascular, anticancer, anti-neurodegenerative; and anti-aging	[81, 84, 90, 96]	
Kaempferol (mg/100 g seed)	155.48 ± 4.34	nd	12 ± 2.00	nd	nd	nd	nd	nd	Anticancer, anticardiovascular, antioxidant, antidiabetic, anti-inflammatory, hepatoprotective, and neuroprotective	[81, 91]	
Catechin (mg/100 g seed)	15 ± 1.5	nd	nd	nd	nd	nd	nd	52.08 ± 1.66	Antioxidant	[81, 97, 98]	
Chlorogenic acid (mg/100 g seed)	16.88 ± 1.14	nd	nd	nd	nd	nd	nd	11.68 ± 0.74	Antidiabetic similar to metformin	[81, 83, 99]	
Cyanidin 3-glucoside (C3G) (mg/100 g seed)	nd	nd	nd	nd	nd	nd	nd	3.16 ± 0.12	Chemopreventive and chemotherapeutic activities of C3G on osteoclast and osteoblast and anticancer	[81, 100]	
Homogentisic acid (mg/100 g seed)	2.63 ± 2.05	nd	nd	nd	nd	nd	nd	11.36 ± 0.54	Inhibit cell growth in vitro/cytotoxic activity against alkaptonuria	[81, 83, 101]	
Tangeretin (mg/100 g seed)	37.67 ± 1.25	nd	nd	nd	nd	nd	nd	nd	Enhances radiosensitivity of GC cells as demonstrated by MTT and colony formation assays	[83, 102]	

*Carotenoids*										
Total carotenoids (*μ*g/100 g seed)	370-790	1164-2669	5690 ± 400	nd	nd	nd	1000-3200	nd	Antioxidant activity by inhibiting fat peroxide	[86, 90, 93, 103, 104]	
Ascorbic acid (mg/100 g seed)	61.22-74.48	7.04 ± 1.76	19.5 ± 0.50	nd	nd	nd	nd	nd	Stopping free radical activity and inhibiting fat peroxide	[86, 104–106]	

*Antiproliferative capacities on cancer cell lines (×100%)* ^∗^										
Lung	0.59 ± 0.03	nd	nd	1.00 ± 0.00	0.75 ± 0.03	0.81 ± 0.03	0.98 ± 0.01	nd	Inhibit the growth of human lung (A549) cancer cell lines	[83]	
Breast	0.48 ± 0.03	nd	nd	1.00 ± 0.02	0.78 ± 0.04	0.49 ± 0.10	0.97 ± 0.01	0.9117	Inhibit the growth of human breast (MCF-7) cancer cell lines	[83, 85]	
Liver	0.41 ± 0.01	nd	nd	0.99 ± 0.02	0.82 ± 0.02	0.66 ± 0.04	0.94 ± 0.00	0.50	Inhibit the growth of human liver (HepG2) cancer cell lines	[83, 85]	
Colon	0.45 ± 0.05	nd	nd	1.00 ± 0.02	0.85 ± 0.03	0.90 ± 0.02	0.98 ± 0.00	nd	Inhibit the growth of human colon (HT-29) cancer cell lines	[83]	

nd: not detected; UDL: under detectable limit. ^∗^Effectively inhibit cell growth at extract ranging from 20 to 100 mg/ml.
